# A Prescribed Chinese Herbal Medicine Improves Glucose Profile and Ameliorates Oxidative Stress in Goto-Kakisaki Rats Fed with High Fat Diet

**DOI:** 10.1371/journal.pone.0060262

**Published:** 2013-04-02

**Authors:** Lin Wu, Xiang Li, Hongguang Zhu, Ping Xu, Xin Gao

**Affiliations:** 1 Department of Endocrinology and Metabolism, Zhongshan Hospital, Fudan University, Shanghai, China; 2 Department of Geriatrics, Zhongshan Hospital, Fudan University, Shanghai, China; 3 Department of Pathology, Shanghai Medical College, Fudan University, Shanghai, China; 4 Shanghai Laboratory Animal Center, Chinese Academy of Science, Shanghai, China; Pennington Biomedical Research Center, United States of America

## Abstract

Oxidative stress (OS) plays a role in hyperglycemia induced islet β cell dysfunction, however, studies on classic anti-oxidants didn’t show positive results in treating diabetes. We previously demonstrated that the prescribed Chinese herbal medicine preparation “Qing Huo Yi Hao” (QHYH) improved endothelial function in type 2 diabetic patients. QHYH protected endothelial cells from high glucose-induced damages by scavenging superoxide anion and reducing production of reactive oxygen species. Its active component protected C2C12 myotubes against palmitate-induced oxidative damage and mitochondrial dysfunction. In the present study, we investigated whether QHYH protected islet β cell function exacerbated by high fat diet (HFD) in hyperglycemic GK rats. 4-week-old male rats were randomly divided into high HFD feeding group (n = 20) and chow diet feeding group (n = 10). Each gram of HFD contained 4.8 kcal of energy, 52% of which from fat. Rats on HFD were further divided into 2 groups given either QHYH (3 ml/Kg/d) or saline through gastric tube. After intervention, serum glucose concentrations were monitored; IPGTTs were performed without anesthesia on 5 fasting rats randomly chosen from each group on week 4 and 16. Serum malondialdehyde (MDA) concentrations and activities of serum antioxidant enzymes were measured on week 4 and 16. Islet β cell mass and OS marker staining was done by immunohistochemistry on week 16. QHYH prevented the exacerbation of hyperglycemia in HFD feeding GK rats for 12 weeks. On week 16, it improved the exacerbated glucose tolerance and prevented the further loss of islet β cell mass induced by HFD. QHYH markedly decreased serum MDA concentration, increased serum catalase (CAT) and SOD activities on week 4. However, no differences of serum glucose concentration or OS were observed on week 16. We concluded that QHYH decreased hyperglycemia exacerbated by HFD in GK rats by improving β cell function partly via its antioxidant effect.

## Introduction

Progressive islet β cell dysfunction is one of the two fundamental pathogenic features of type 2 Diabetes Mellitus (T2D). Islet β cell dysfunction is now considered to be essential for the development of T2D, since its insufficient compensation leads to the onset of glucose intolerance [1] and its progressive exacerbation explains the unmet needs of long-term glycemic control [2], [3]. Accumulating evidence confirmed that islet β cell dysfunction, manifested by secretory incapability and reduced β cell mass, is associated with increased oxidative stress (OS) induced by over-nutrition, hyperglycemia, dyslipidemia and chronic inflammation [4], [5], [6], [7]. It has been confirmed that total antioxidant status in diabetic patients is markedly low [8], [9], and the intrinsic anti-oxidative defence system of islet β cell is fragile [10]. Therefore, antioxidant might be one promising auxiliary strategy to delay β cell failure. Since clinical researches on classic anti-oxidants did not show promising results [8], [11], [12], [13], it is suggested by Ceriello et al [14] that new antioxidant, either regulates free radical over-production at the mitochondrial level or increases intracellular anti-oxidant defence system, might be the key to link the theoretical possibility of antioxidant therapy to its practical use.

QHYH is a traditional Chinese medicine (TCM) formula. In a single-blinded, randomized, controlled clinical trial conducted in 2004, QHYH reduced urinary albumin excretion rate (UAER) and ameliorated micro-circulation of nail bed flow in type 2 diabetic patients, and the protective effect was independent of serum blood glucose concentration and lipid control, indicating that QHYH could improve endothelial function [15]. Since gene chip analysis showed that QHYH mainly regulated oxidative phosphorylation and glutathione pathways, we then measured the free radical scavenging ability of QHYH using electron paramagnetic resonance (EPR) and found that QHYH scavenged hydroxyl radical generated in the DMPO/HO⋅ adduct generating system and superoxide anions in endothelial (bEnd.3) cells incubated in high glucose (35 mmol/L) medium [16]. We further confirmed that both QHYH and its active ingredient tetramethylpyrazine (TMP) protected endothelial cells from high glucose induced damages by reversing ROS production, down-regulating Akt/eNOS phosphorylation and reducing NO generation [17]. In addition, TMP protected C2C12 myotubes against palmitate-induced oxidative damage and mitochondrial dysfunction, and reversed its suppression on insulin-stimulated glucose uptake in muscle cells partially through up-regulation of mitochondrial biogenesis [18].

Based on the fact that QHYH protected endothelial cells and muscle cells against glucose- and palmitate-induced OS by down-regulating mitochondrial ROS production, we hypothesized that QHYH might also protect islet β cell against hyperglycemia induced OS by decreasing the over-production of ROS and help to maintain glucose control in diabetic individuals. In this setting, we chose non-obese spontaneous Goto-Kakasaki (GK) rats,that share many similarities in islet phenotype with type 2 diabetic patients [19] and are confirmed to have elevated systemic and pancreatic OS [20],as subjects. Before weaning, hyperglycemia does not appear in GK rats and can be considered pre- diabetic. We fed 4-week old male GK rats with high fat diet to exacerbate hyperglycemia and magnify OS, and simultaneously treated them only with QHYH, in order to determine whether QHYH improves glucose metabolism by mitigating OS exacerbated by high fat diet.

## Materials and Methods

### Ethics Statement

This study was performed in strict accordance with the recommendations in the Guide for the Care and Use of Laboratory Animals of the National Technical Department. All animal experiments were conducted with the approval of the Institutional Animal Care and Use Committee (IACUC) of Zhongshan Hospital, Fudan University. Surgery was performed under chloral hydrate anesthesia, and every effort was made to minimize suffering.

### Diets and Drug

Chow diet (M02-F, each 100gram contained 320 Kcal of calorie, 30% of which from protein, 15% from fat, and 55% from carbohydrate) and high-fat diet (each 100gram contained 480 Kcal, 22% of which from protein, 52% from fat, and 26% from carbohydrate) were purchased from Slac Lab Animal Co. (Shanghai).

QinghuoYihao (QHYH) was kindly donated by the pharmacies of Zhongshan hospital where it was produced according to a standard way of preparing traditional herbal medicine [16], [17].

### Animals

4-week-old male GK rats, purchased from Slac Laboratory Animal Center, were randomly divided into 2 dietary groups: high fat diet group (HFD, n = 20) and chow diet group ((ND, n = 10). Rats on HFD were further divided into 2 groups (n = 10 each) given either QHYH (3 ml/Kg/d) or saline (3 ml/Kg/d) through gastric tube twice a day with an interval of 8 hours. All rats had free access to food and sterile tap water *ad libitum*. For 16 weeks, all the rats were kept in specific pathogen-free rooms controlled for temperature (22–26°C), humidity (40%–70%) and 12-hour light/dark light cycle at the animal center of Chinese Academy of Science.

### Experimental Protocol

Body weight and food consumption were recorded weekly at fixed time. During the 16-week study, 0.5∼1 mL of blood samples after a 14-hour overnight fast were collected every week from the retro-orbital plexus for 12 weeks and every 2 weeks from week 12 to week 16 for measurement of serum glucose concentrations and liver/renal function. From week 1 to week 10, 0.5∼1 mL non-fasting blood samples were collected at fixed time (2pm every Wednesday) from retro-orbital plexus for measurement of serum glucose concentrations. Serum alanine transaminase (ALT), aspartate aminotransferase (ALT), blood urea nitrogen (BUN), creatinine (Cr), as well as malondialdehyde (MDA) concentrations and activities of antioxidant enzymes were measured using fasting blood samples. Glycosylated serum protein (GSP) concentrations were measured using feeding blood sample. All blood samples were immediately centrifuged at 4°C and serum was kept in −70°C fridge.

Serum glucose concentration was determined by hexokinase method using reagents from Sysmex (Jinan, China). GSP concentrations, as well as serum ALT, AST, BUN and Cr concentrations, were measured using commercial diagnostic kits (Sysmex, Jinan, China) following manufacturer instructions using Hitachi 7020 clinical analyzer (Hitachi, Japan). Serum MDA concentration was determined using commercial kits with spectrophotometric (BIO-RAD, Japan) measurement of the color produced during the reaction to thiobarbituric acid (TBA) at 532 nm. Activity of serum SOD was measured by xanthine oxidase method using commercial kits with the absorbance measured using spectrophotometer at 550 nm. Activities of serum CAT and GSH-Px were measured by colorimetric method measuring absorbance at 405 nm and 412 nm respectively using spectrophotometer. All the assay kits (MDA, CAT, SOD and GSH-Px) were purchased from Jiancheng Bioengineering Institute (Nanjing, China).

### Intraperitoneal Glucose Tolerance Tests (IPGTTs)

Glucose tolerance tests were done on week 4 and week 16 after intervention. 5 rats were randomly chosen from each group. No anesthetics were used during the tests. After a 14-hour overnight fast, 1∼1.5 ml of fasting blood samples were drawn from the retro-orbital plexus for measurement of fasting serum glucose and insulin concentrations. Then 20% D-glucose (2 g/Kg body weight) was injected intraperitoneally. Around 0.8 ml of blood sample was drawn from the retro-orbital plexus at 15, 30, 60, and 120 minutes afterwards for measurement of serum insulin and glucose concentration. Blood samples were centrifuged immediately at 3,000 rpm for 10 min at 4°C. Serum insulin concentrations were measured using the Sensitive Rat Insulin RIA kit (Linco Research).

Area under the curve (AUC) of serum glucose and insulin concentrations from 0 to 2 hours during IPGTT was calculated as:

AUCglucose(0–2 h) = 1/8(G0+G15)+1/8(G15+G30)+1/4(G30+G60)+l/2(G60+G120)

AUGinsulin(0–2 h) = 1/8(I0+I15)+1/8(I15+I30)+1/4(I30+I60)+l/2(I60+I120)

Ratio of AUCinsulin to AUCglucose = AUCinsulin/AUCglucose

### Histological Examination

After a 14-hr overnight fast on week 16, rats were fully anaesthetized by intraperitoneal injection of chloral hydrate (10 mg/Kg). After 4–5 ml blood collected from the descending aorta, pancreas were carefully excised, washed in saline, trimmed of adipose tissue, blotted, weighed and fixed in 4% paraformaldehyde overnight and embedded in paraffin. After locating the epididymis, adipose tissue around the organ was completely taken from both sides and weighed according to the descriptions of Cinti [21].

5 pancreata were randomly chosen from each group. Each pancreas was longitudinally cut into 6 µm thick sections serially by microtome. To avoid bias from regional changes in islet distribution and islet cell composition, 4 consecutive sections were chosen from 5 evenly divided transects throughout the block. Altogether 20 sections were chosen. Sections from each transects were coded as No1 to No4.

All the fixed tissues were dehydrated through graded ethanol series and made transparent with xylene for hematoxylin/eosin (using sections coded as No1) and immunohistochemical staining. For quantification of pancreatic islet size, sectional areas of pancreas were analyzed with an colored cell image software (IMS, Shenteng Information & Technology Co.Ltd, Shanghai, China). Pictures were taken using a digital camera (Panasonic MV-CP410, Panasonic, Japan).

### Immunohistochemistry

Immunohistochemical staining was done on sections coded No 2 to No4, using insulin, nitrotyrosine (NT) and 8-Hydroxy-2′deoxyguanosine (8OHdG) as primary antibody respectively. Immunohisto-staining was done strictly following the instructions of manufacturer. In short, de-paraffinized and dehydrated sections were treated in a microwave oven at low power for 10 min in 0.01 M sodium citrate buffer (pH 6.0) to retrieve antigen, and inactivated endogenous peroxidase using 3% hydrogen peroxide in methanol for 10 min, and blocked with blocking solution for 1 hour at room temperature. Sections were then incubated respectively with, 1) 1∶400 dilution (diluted in blocking solution) of monoclonal antibody to insulin (insulin C27C9 Rabbit mAb #3014, Cell Signaling Technology Inc, MA, USA) overnight at 4°C; 2) 1∶20 dilution of mouse monoclonal antibody to 8-hydroxy-2′-deoxyguanosine (8OHdG) (ab48508, Abcam, Cambridge, UK) overnight at 4°C; 3) with a 1∶100 dilution of mouse anti-nitrotyrosine monoclonal antibody (MAB5404, chemicon, CA, USA) for 10 min at room temperature. Incubation of biotinylated anti-rabbit and anti-mouse second antibody and HRP-labeled Streptavidin, and staining with DAB was done according to the product instructions of the manufacturer (anti-mouse: LHK611/anti-rabbit: LHK 612, Histostain-Plus Kit, Jingmei Biotech, Shanghai, China) using LAB-SA technology and counterstained with haematoxylin.

After staining, sections were then examined using Colored Cell Image software (IMS, Shenteng Information & Technology Co.Ltd,Shanghai, China). Of each section, the percentage of islet area out of pancreas area was calculated by dividing the sum-up of all the islet area by the whole pancreas area measured under light microscope with ×100 magnificence. The percentage of β cell area in islet was calculated by dividing the sum-up area of all insulin-positive area by islets area in each section. β cell mass was calculated by multiplying the pancreas weight (mg) by the percentage of β cell area out of islet area (%) and the percentage of islet area out of pancreas area (%) [22]. To semi-quantitatively calculate insulin content, we measured gray level of insulin staining positive area. Gray level refers to the brightness of a pixel, and the value representing the lightness from black to white is usually defined as a value from 0 to 255, with 0 being black and 255 being white. Grey level was measured for 5 randomly chosen non-overlapping islets on sections stained with insulin of each sample. Altogether 125 islets were measure and the arithmetic average was calculated as the mean gray level.

Also, 5 non-overlapping islets were randomly chosen from each section to measure the positive staining area of NT and 8OHdG and the corresponding islet area.

### Statistical Analysis

Data were expressed as mean±SEM. Statistical analysis was performed using SPSS12.0 with one-way analysis of variance (ANOVA) followed by Turkey’s analysis. A difference was defined as significant when *P*<0.05.

## Results

Altogether 30 rats were studied. Only 19 rats survived (7 from group ND, 6 from group HFD and 6 from group HFD-QH) after 16 weeks of observation (Table 1). The survival rate was comparable among the groups. From each group, 2 out the 5 rats that underwent IPGTT died afterwards on week 4 because of loss of blood. The high death rate might be attributed to the frequency and volume of blood drawing. Furthermore, with the prolongation of high fat diet, not only hyperglycemia was exacerbated which caused more infection, but also injuries caused by frequent blood taken, led to more death of the rats. Therefore monitor of feeding serum glucose concentration discontinued from week 10 on. Monitor of fasting serum glucose concentration was reduced to every 2 weeks from week 12 to week 16.

**Table 1:** Number of rats survived at different time after intervention in each group.

	week 4	week 8	week 12	week16
ND	8	7	7	7
HFD	8	6	6	6
HFD-QH	8	7	6	6

### QHYH Increased Body Weight of GK Rats Fed with High Fat Diet

**Figure 1:**
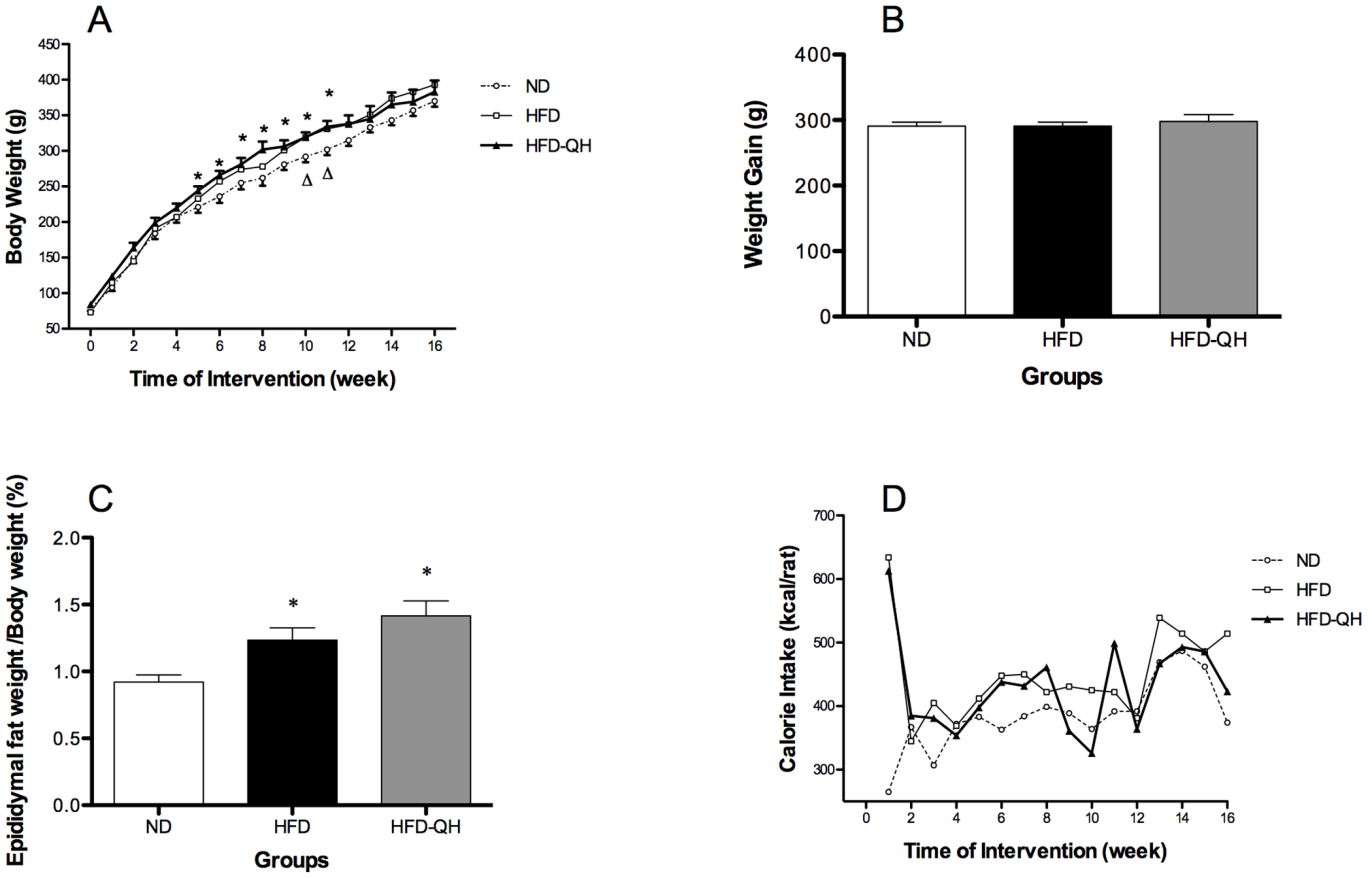
QHYH further increased body weight of GK rats fed with high fat diet. Body weight of rats from each group (A), body weight gain after 16 weeks of intervention (B), ratio of epididymal fat to body weight after 16 weeks of intervention (C) and the average calorie intake of each rat from different groups (D) were presented. ND: normal diet; HFD: high fat diet; HFD-QH: high fat diet plus 3 ml/Kg/day QHYH given via gastric tube twice a day. Values and bars represent means±SEM at each time point. *denote *P*<0.05 for chance differences versus group ND. △ denotes *P*<0.05 versus group HFD.

Mean caloric intake was similar among 3 groups except for the first week (Fig. 1D). Rats of group HFD were only markedly heavier than rats of group ND on week 10 and week 11 after intervention. QHYH further increased body weight of GK rats that from week 5 to week 11, rats of group HFD-QH were heavier than those of group ND (*P*<0.05)). No significant differences of body weight were observed among 3 groups from week 12 on and between the two high fat feeding groups at any time point (Fig. 1A). After 16 weeks of intervention, body weight gain was not statistically different among 3 groups (Fig. 1B), however, the ratio of epididymal fat weight to body weight was markedly increased in the two high fat feeding groups (Fig. 1C).

### QHYH Decreased the Exacerbated Hyperglycemia in GK Rats Fed with High Fat Diet

**Figure 2:**
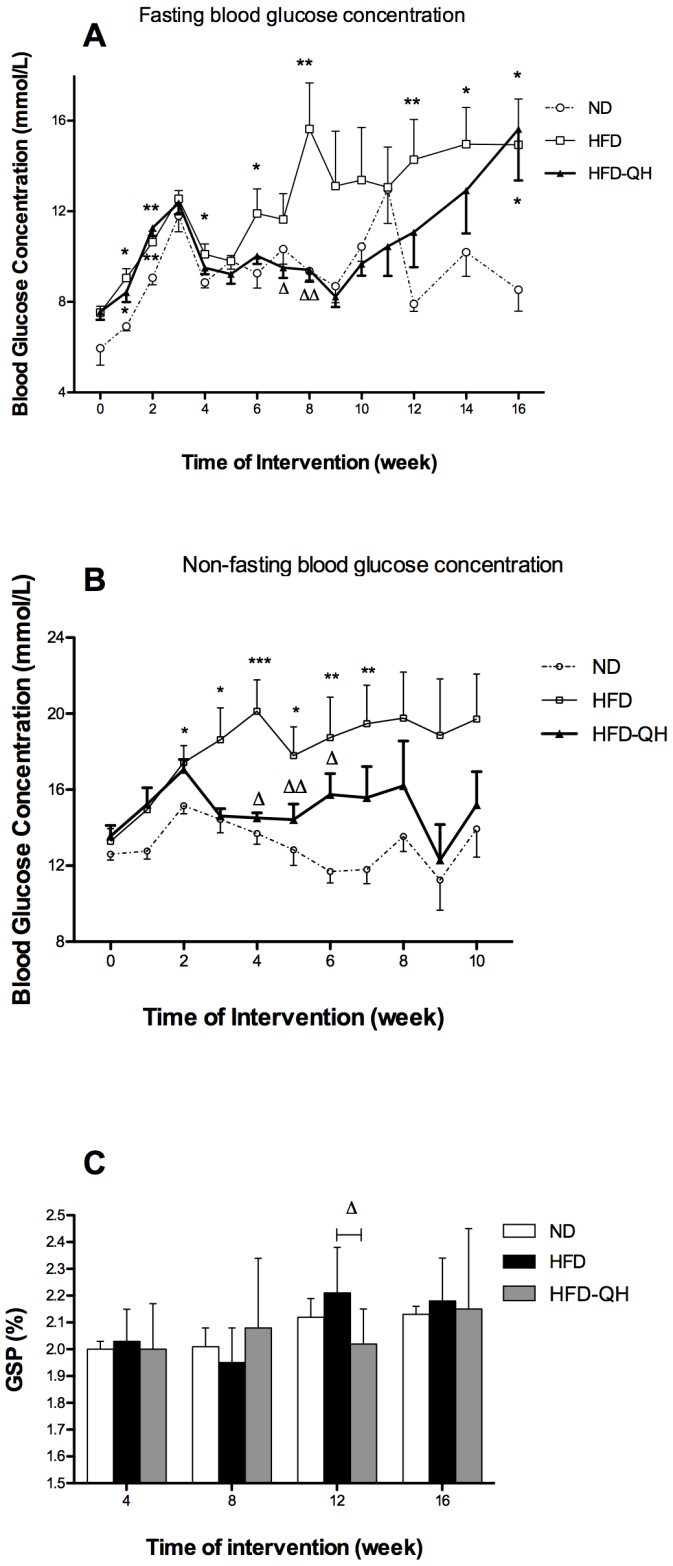
QHYH decreased hyperglycemia exacerbated by high fat diet in GK rats. Fasting serum glucose concentrations of each group during 16 weeks of intervention (A), feeding serum glucose concentrations of each group from the beginning to week 10 of the study (B), and glycosylated serum proetin concentrations of each group measured every 4 weeks (C) were presented. Values and bars represent means±SEM at each time point. *, ** and *** denote *P*<0.05, *P*<0.01 and *P*<0.001 respectively versus group ND. △ and △△ denote *P*<0.05 and *P*<0.01 versus group HFD.

GK rats became diabetic at the age of 5 weeks (1 week after intervention) that fasting blood concentration exceeded 7 mmol/L. Rats of group HFD manifested higher fasting serum glucose levels than group ND and the differences were significant on week 1, 2, 4, 6, 8, 12, 14 and 16 after intervention. Glucose concentrations of group HFD-QH became lower than those of group HFD from week 4 on, and the difference was statistically significant at week 7 and week 8. Differences of fasting serum glucose concentrations between group HFD-QH and group ND were not statistically significant, except for week 1,2 and 16 (Fig. 2A). Feeding serum glucose concentrations of group HFD were higher than those of group ND, and the differences were statistically significant from week 2 to week 7. Feeding serum glucose concentrations were significantly lower in group HFD-QH than in group HFD from week 4 to week 6 after intervention. There was no marked difference between group HFD-QH and group ND throughout the monitoring period (Fig. 2B). Serum GSP level was markedly lower in group HFD-QH than in group HFD on week 12 after intervention (Fig. 2C).

### QHYH Improved the Exacerbated Glucose Tolerance in GK Rats Fed with High Fat Diet

**Figure 3:**
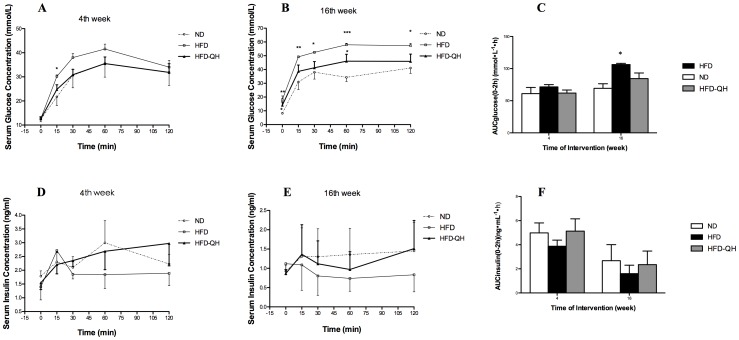
QHYH improved the exacerbated glucose tolerance induced by high fat diet in GK rats. Intraperitoneal glucose tolerance tests (2 mg/Kg body weight of glucose) were performed on rats randomly chosen from each group. Tests were done without anesthetics. Serum glucose concentrations during IPGTT of each group after 4 weeks of intervention (A) and 16 weeks of intervention (B), and area under the curve of different groups (C) were presented. Serum insulin concentrations during IPGTT of each group after 4 weeks (D) and 16 weeks of intervention (E), and area under the curve of different groups (F) were also presented. Values and bars represent means±SEM for 5 rats per group. *, ** and *** denote *P*<0.05, *P*<0.01 and *P*<0.001 respectively versus group ND.

Except for glucose concentration at 15 min after glucose load, no significant difference of either glucose concentrations at any time point or AUC of glucose concentrations was observed among the 3 groups on week 4 after intervention (Fig. 3A&C). On week 16, glucose tolerance of group HFD was markedly decreased compared to that of group ND that both serum glucose concentrations at each time point (Fig. 3B) and AUCglucose (Fig. 3C) of group HFD were markedly higher than those of group ND. The difference between group HFD-QH and group ND was not statistically significant. Both serum insulin concentrations at each time point after glucose load and AUCinsulin after glucose load of group HFD-QH tended to be increased to near to the level of group ND, though statistically insignificant (Fig. 3D∼F).

### QHYH Prevented the Further Loss of β Cells Mass in GK Rats Fed with High Fat Diet

**Figure 4:**
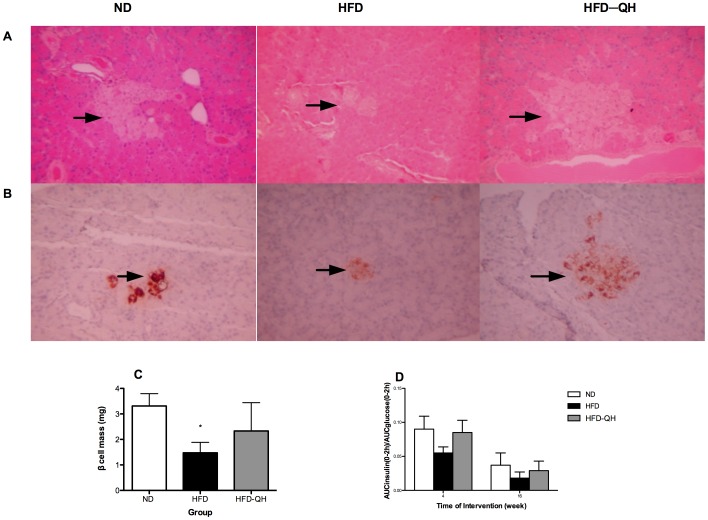
QHYH prevented the further loss of β cells mass induced by high fat diet. Pictures of haematoxylin and eosin staining of pancreas of GK rats from each group (A) and insulin immunohistochemical staining of pancreas of GK rats from each group (B) were presented. An islet is encircled in each quadrant. Black arrows point to islets of Langerhans. (Original magnification ×200). Islet β cell mass calculated by multiplying the pancreas weight (mg) by the percentage of β cell area out of islet area (%) and the percentage of islet area out of pancreas area (%) was presented (C). Ratio of AUCinsulin to AUCglucose (0–2 h) after 4 weeks and 16 weeks of intervention was shown (D). n = 5 in each group. Values and bars represent means±SEM. * denotes *P*<0.05 versus group ND.

The islets of GK rat were irregular in shape. The boundary between the islet and exocrine pancreas was broken and cells were degenerated and swollen. Islets from group HFD showed further atrophy and reduced cell number inside islets when compared to group ND. QHYH partly repaired the injury by increasing both islet cell number and islet area to near the level of group ND (Fig. 4).

For immunohistochemical staining, the insulin expression was brown granule in cytoplasm (Fig. 4B). Mean gray level of insulin positive staining of group HFD was significantly greater than that of group ND (125.52±27.39 vs 93.41±14.17, *P*<0.05), and was decreased in group HFD-QH (105.60±15.24). No difference was observed between group HFD-QH and group ND. There was a marked 55.3% reduction of β cells mass in pancreas of rats from group HFD compared to that of group ND, which was partly restored in group HFD-QH (Fig. 4C). Though of no statistical significance, ratio of AUCinsulin to AUCglucose of group HFD was less than half of that of group ND, and was brought back to near the level of group ND in group HFD-QH on week 4 and week 16 (Fig. 4D).

### QHYH Decreased OS Marker in GK Rats Fed with High Fat Diet Only after 4 Weeks of Intervention

**Figure 5:**
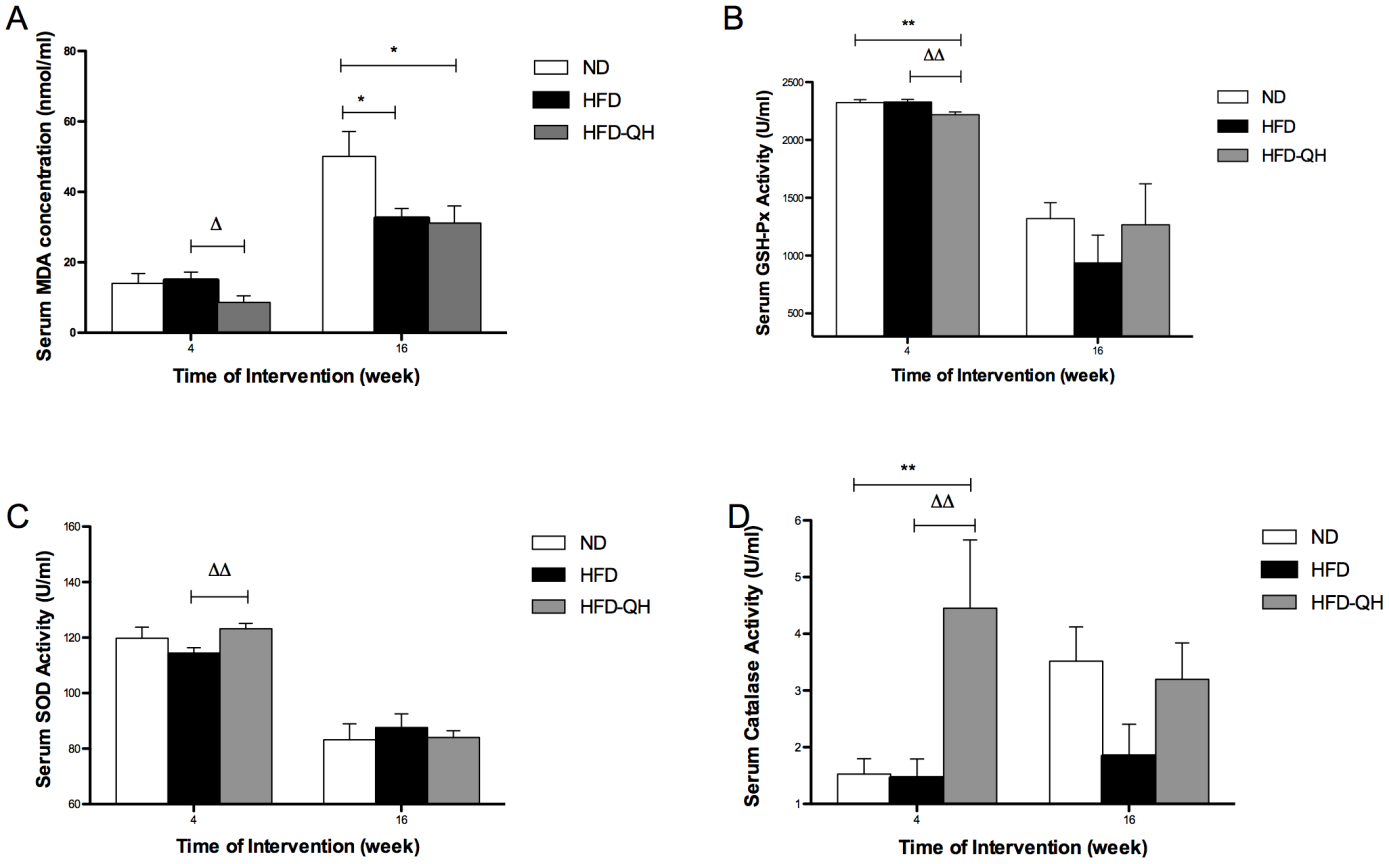
QHYH alleviated oxidative stress in GK rats fed with high fat diet only after 4 weeks of intervention. Serum malondialdehyde (MDA) concentrations (A), and activities of serum anti-oxidant enzyme glutathione peroxidase (GSH-Px) (B), SOD (C), and catalase (CAT) (D) of each group were measured using fasting blood sample taken on week 4 and week 16 after intervention respectively. Values and bars represent means±SEM at each time point. There were 10 rats from each group on week 4 after intervention. On week 16, there survived 7 rats in group ND, 6 rats in group HFD, and 6 in group HFD-QH. * and ** denote *P*<0.05 and *P*<0.01 respectively compared versus group ND. △ and △△ denote *P*<0.05 and *P*<0.01 respectively versus group HFD.

On week 4, serum MDA concentration of group HFD-QH was significantly lower than that of group HFD. However, after 16 weeks of intervention, no significant difference was observed between the two high fat feeding groups (Fig. 5A). When compared to group HFD, QHYH markedly increased the activities of both SOD and CAT, but decreased the activity of GSH-Px on week 4, while on week 16, no differences of either anti-oxidant enzyme existed among the groups (Fig. 5B–D).

**Figure 6:**
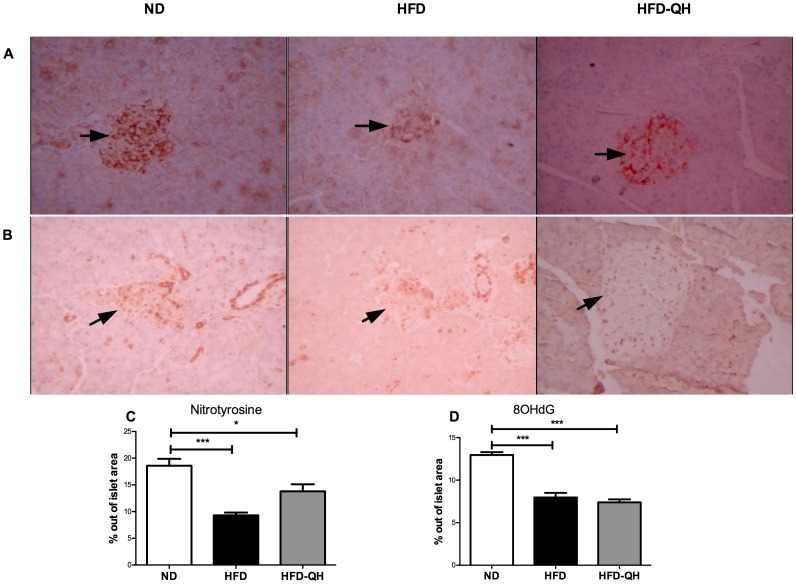
QHYH had no effect on oxidative products inside islets after 16 weeks of intervention. Immunohistochemical staining of nitrotyrosine (A) and 8-hydroxy-2′-deoxyguanosine (B) was performed on 5 rats randomly chosen from each group. An islet is encircled in each quandrant. Black arrows point to islets of Langerhans (Original magnification ×200). Percentage of immunohistochemial positive staining area in islet out of the area of corresponding islet (C, D) was presented. Values and bars represent means±SEM. * and ** denote *P*<0.05 and *P*<0.001 versus group ND.

Nitrotyrosine (NT, Fig. 6A) and 8OHdG (Fig. 6B) positive staining cells were present both inside and outside islets of GK rats. Compare to group ND, percentages of NT and 8OHdG positive labeling area out of islet area were markedly lower in both group HFD and group HFD-QH, but were not different between the 2 high fat feeding groups. On the contrary, markedly-positive cells localized at the islet periphery were largely reduced by the intervention of QHYH (Fig. 6).

### QHYH Prevented Liver Dysfunction Induced by High Fat Diet in GK Rats

Changes of liver and renal function were shown in Table 2. Serum ALT concentrations of group HFD were higher than those of group ND both on week 4 and week 16 after intervention. No significant differences of serum ALT concentrations were observed between group HFD-QH and group ND. On week 4, serum AST concentration of group HFD-QH was markedly lower than that of group ND; serum BUN concentration of HFD group was significantly lower than that of group ND. On week 16, serum BUN concentration of group HFD-QH was markedly lower than that of group HFD. No significant difference of serum creatinine concentration was observed among 3 groups.

**Table 2:** Changes of liver and renal function of GK rats fed with high fat diet.

		ND	HFD	HFD-QH
4^th^ week	ALT(IU/L)	83.1±4.79	96.40±4.18*	94.00±3.16
	AST(IU/L)	143.8±9.77	123.70±6.10	116.50±5.07*
	BUN(mmol/L)	5.4±0.17	3.15±0.13^***^	3.39±0.20^***^
	Cr (µmol/L)	63.4±30.31	17.20±1.33	19.60±5.50
16^th^ week	ALT (IU/L)	74.7±5.60	104.00±9.77*	89.33±7.72
	AST(IU/L)	173.9±11.85	178.44±11.57	156.67±5.66
	BUN (mmol/L)	6.6±0.23	6.46±0.32	4.78±0.28^**##^
	Cr(µmol/L)	46.7±1.74	45.40±2.21	45.77±2.51

Serum ALT, AST, BUN and creatinine concentrations were measured using commercial kits on Hitachi 7020 clinical analyzer. Values represent means±SEM for 10 rats in each group on week 4 after intervention. On week 16, there left 7 rats in group ND, 6 in group HFD and 6 in group HFD-QH.

* , ** and *** denote *P*<0.05, *P*<0.01 and *P*<0.001 versus group ND.

## denotes *P*<0.01 versus group HFD.

ALT: Alanine Transaminase; AST: Aspartate Aminotransferase; BUN: Blood Urea Nitrogen; Cr: Creatinine. ND: normal diet; HFD: high fat diet; HFD-QH: high fat diet plus QHYH 3 ml/Kg/day.

## Discussion

The main findings of the present study were that the prescribed traditional Chinese medicine preparation QHYH partly reversed islet β cell dysfunction exacerbated by high fat diet in hyperglycemic GK rats that it prevented the elevation of blood glucose concentrations induced by high fat diet for 12 weeks, improved the exacerbated glucose tolerance and prevented the further loss of islet β cell mass induced by high fat diet after 16 weeks of intervention. The protection effect can partly be explained by its *in vivo* antioxidant effect that QHYH decreased serum MDA concentration and increased activities of serum anti-oxidant enzymes in GK rats fed with high fat diet after 4 weeks of intervention.

Even though islet β cell dysfunction plays a key role in the onset and development of diabetes [23] and its unrelenting deterioration explains the failure of long-term glycemic control, few of the currently available anti-diabetics targeted improving β cell function [3]. We found in our study that after 16-weeks of intervention, glucose tolerance was improved, and further loss of islet β cell mass was prevented in group HFD-QH. Also, serum insulin concentrations tended to be increased to near to level of group ND, though statistically insignificant with only 5 rats in each group. The changes of serum insulin concentrations were consistent with the increase of both islet β cell mass and insulin content, reflecting that the glucose lowering effect of QHYH on GK rats fed with high fat diet might be attributed to its protection on islet β cell function. Ceriello et al [24] suggested that 2 pathways exist in the hyperglycemia induced endothelial dysfunction, that neither anti-diabetic nor anti-oxidant alone was able to normalize endothelial function. Since excess OS plays a major role in islet β cell deterioration that it links chronic hyperglycemia [25], [26] and several other toxics [27], [28], [29] to β cell dysfunction, the same pathways might be simultaneously activated. In the present study, QHYH manifested anti-oxidant effect on week 4, and lowered blood glucose concentrations within 12 weeks. With the exceeding duration of high fat diet, hyperglycemia was exacerbated which chronically induced over-production of ROS that exceeds the ROS scavenging effect of QHYH, that after 16 weeks of high fat diet, blood glucose concentrations markedly increased and were comparable in the 2 high fat feeding groups, indicating the protection of QHYH disappeared. Take into account that no anti-diabetic agents were used in our study, our results might be of significant clinical importance that early intervention with a combination of anti-oxidants and anti-diabetic agents might slow down or even reverse the natural progressive course of islet β cell dysfunction.

The underlying mechanisms that QHYH protected islet β cell function awaits further exploration, but might be related to its ability to reverse glucose- and palmitate-induced mitochondrial ROS production [17], [18]. We previously confirmed in 3 different types of endothelial cells that QHYH completely abrogated high glucose-induced ROS production to a level that was well below the baseline, displaying a comparable effect as the conventional antioxidant NAC (N-acetyl-cysteine), and reversed high glucose-induced suppression of NO generation and eNOS (Ser-1177)/Akt (Ser-473) phosphorylation. TMP, the active component of QHYH, blocked drops in mitochondrial membrane potential caused by palmitate, modulated mitochondrial DNA replication and biogenesis, restored oxygen consumption and mitochondrial respiratory chain protein complex III levels. The above-mentioned results suggested that QHYH is capable of protecting against hyperglycemia- and dyslipidemia- induced mitochondrial dysfunction. Whether QHYH protected islet via the same pathways will be focus of our future investigation. Furthermore, the stimulation of glucose uptake by muscle cells observed in our previous study [18] might be another mechanism that increased insulin sensitivity.

We here confirmed previous findings [30], [31] that high fat diet induced minimal changes in body weight of GK rats. Calorie intake was comparable among the groups except for the first week, indicating that the composition of diet, rather than whole calorie consumption, might be the decisive factor of diet-induced obesity in this spontaneous T2D rodent model with genetic defects. At the end of the study, the ratio of epididymal fat to body weight was significantly increased in the 2 high fat feeding groups compared to group ND. Epididymal fat is one part of white adipose tissue (WAT), where excess energy intake is stored in the form of triglyceride [21] and is generally used to represent ‘visceral fat’ in rodents [30], however, biological features and measurement [32] of epididymal fat might not be the same as ‘visceral fat’ which is considered to be closely related to increased insulin resistance [4] and to be the root abnormality of metabolic syndrome that consists of several metabolic disorders including elevated serum triglyceride (TG) level [33]. We observed in our study that when epididymal fat weight was significantly increased in group HFD-QH than group ND after 16 weeks of intervention, serum TG concentration was not increased (data not shown). Therefore, the real effect of QHYH on epididymal fat in GK rats would need further exploration.

Inconsistent with previous findings that over-nutrition is related to heightened systemic OS [4], [34], [35], [36], [37] and dietary restriction ameliorates systemic OS [38] in GK rats, we found that after 16 weeks of high fat diet, serum MDA concentration, as well as nitrotyrosine and 8OHdG deposits inside islets, was significantly lower in high fat feeding GK rats than in normal diet feeding GK rats. The underlying mechanisms of the discrepancies were unknown, and might be explained by variations in lipid composition of diet, or the age of GK rats when high fat diet was given, or variable strains of GK rats. Despite the stable degree of glucose intolerance, other characteristics of GK rats might be different between various strains due to local breeding environment [19]. Lacraz et al [20], [39] found that in GK rats, elevated ROS levels were present in islet in pre-diabetic stage and OS marker (8-OHdG and 4-hydroxy-2-nonenal) positive cells were predominantly localized at the islet periphery or along ducts, not in endocrine cells within the core of the islets. After the onset of diabetes, islet cells develop an unexpected adaptive protection to counteract chronic OS by increasing anti-oxidant defense system to maintain basal ROS accumulation lower than that in non-diabetic islet cells. These islets are less sensitive to exogenously applied ROS, suggesting that up-regulation of antioxidant defense is part of the intrinsic mechanisms for self-protection against hyperglycemia in diabetic GK rats. Additionally, the markers we chose here are the products of oxidative stress that reflect only part of the total oxidative status in vivo. There might exist more sensitive and specific markers of oxidative stress that we did not measure.

We observed here that QHYH prevented liver dysfunction induced by high fat diet in GK rats. Anti-oxidants are generally used to treat liver dysfunction clinically, therefore, it is conceivable that anti-oxidant QHYH protected against liver dysfunction induced by high fat diet.

In conclusion, intervention with QHYH prevented the exacerbation of hyperglycemia in high fat feeding GK rats within 12 weeks. Also, QHYH treatment prevented the exacerbation of glucose tolerance, further loss of islet β cell mass and decrease of insulin content induced by high fat diet after 16-weeks of intervention. After 4 weeks of treatment, QHYH presented *in vivo* anti-oxidant effect that serum MDA concentration was markely decreased, and activities of serum anti-oxidant enzymes CAT and SOD were significantly increased in high fat feeding GK rats. The beneficial glucose lowering effect of QHYH might be mediated by improving β cell function via its *in vivo* antioxidant effect and might be limited to a certain period of time that protective effect of QHYH was compromised by prolonged chronic hyperglycemia. Therefore, we suggest that early intervention with a combination of anti-oxidant and anti-diabetic agents might help to maintain long-term glycemic control.
